# Laboratory-confirmed pandemic H1N1 influenca in hospitalized adults – findings from the Canadian Nosocomial Infection Surveillance Program (CNISP), 2009-10

**DOI:** 10.1186/1753-6561-5-S6-P81

**Published:** 2011-06-29

**Authors:** G Taylor, K Wilkinson, D Gravel, B Amihod, C Frenette, D Moore, A McGeer, K Suh, A Wong, R Mitchell, Canadian Nosocomial Infection Surveillance Program

**Affiliations:** 1 Univesity of Alberta, Edmonton, Canada; 2 Public Health Agency of Canada, Ottawa, Canada; 3 Jewish General Hospital, Canada; 4 McGill University, Montreal, Canada; 5 University of Toronto, Toronto, Canada; 6 University of Ottawa, Ottawa, Canada; 7 University of Saskatchewan, Saskatoon, Canada

## Introduction / objectives

To describe laboratory-confirmed pandemic H1N1 (pH1N1) influenza in adult inpatients at participating Canadian hospitals between June 2009 and May 2010 and compare to previous years’ seasonal surveillance.

## Methods

Adult inpatients (>=16 years) with lab confirmed influenza were enrolled. Variables collected included ICU admissions and death attributed to influenza assessed 30 days after initial diagnosis.

## Results

### Results

Thirty-seven hospitals submitted data on 701 cases**.** The median age of was 49 years (range 16 - 94). Vaccine history was available for 314 cases, and 21% (n=65) reported receiving vaccine. Oseltamivir was given to 90% of the cases a median of 3 days after symptom onset (range 0 - 24). Influenza-associated admission to ICU was required for 28% (n=197). The 30 day all-cause mortality was 7%; influenza was the primary cause of 20 deaths and contributed to death in a further 22 cases for an influenza-attributed mortality of 6%. The mean age at death was 50 years (SD 13.8).

## Conclusion

The ICU admission rate and influenza-attributed mortality were similar to three preceding influenza years, but mean age at death was significantly younger (p<0.01). Antivirals were prescribed for more patients with influenza (90%) than in previous seasons (35-47%). The pH1N1 virus appeared outside the traditional influenza season and impacted a different age group than seasonal viruses circulating in previous years, highlighting the importance of ongoing influenza surveillance.

## Disclosure of interest

None declared.

